# Hypoglycaemia due to insulin therapy for the management of hyperkalaemia in hospitalised adults: A scoping review

**DOI:** 10.1371/journal.pone.0268395

**Published:** 2022-05-12

**Authors:** Mogamat-Yazied Chothia, Toby Humphrey, Anel Schoonees, Usuf Mohamed Ebrahim Chikte, Mogamat Razeen Davids

**Affiliations:** 1 Division of Nephrology, Department of Medicine, Faculty of Medicine and Health Sciences, Stellenbosch University, Cape Town, South Africa; 2 Division of Experimental Medicine and Immunotherapeutics, Department of Medicine, University of Cambridge, Cambridge, United Kingdom; 3 Centre for Evidence-based Health Care, Division of Epidemiology and Biostatistics, Faculty of Medicine and Health Sciences, Stellenbosch University, Cape Town, South Africa; 4 Department of Global Health, Faculty of Medicine and Health Sciences, Stellenbosch University, Cape Town, South Africa; Stanford University School of Medicine, UNITED STATES

## Abstract

**Introduction:**

Hyperkalaemia is a very common electrolyte disorder encountered in hospitalised patients. Although hypoglycaemia is a frequent complication of insulin therapy, it is often under-appreciated. We conducted a scoping review of this important complication, and of other adverse effects, of the treatment of hyperkalaemia in hospitalised adults to map existing research on this topic and to identify any knowledge gaps.

**Materials and methods:**

We followed the PRISMA-ScR guidelines. Studies were eligible for inclusion if they reported on any adverse effects in hospitalised patients ≥18-years-old, with hyperkalaemia receiving treatment that included insulin. All eligible research from 1980 to 12 October 2021 were included. We searched Medline (PubMed), Embase (Ovid), the Cochrane Library, CINHAL, Africa-Wide Information, Web of Science Core Collection, LILACS and Epistemonikos. The protocol was prospectively registered with the Open Science Framework (https://osf.io/x8cs9).

**Results:**

Sixty-two articles were included. The prevalence of hypoglycaemia by any definition was 17.2% (95% CI 16.6–17.8%). The median timing of hypoglycaemia was 124 minutes after insulin administration (IQR 102–168 minutes). There were no differences in the prevalence of hypoglycaemia when comparing insulin dose (<10 units vs. ≥10 units), rate of insulin administration (continuous vs. bolus), type of insulin (regular vs. short-acting) or timing of insulin administration relative to dextrose. However, lower insulin doses were associated with a reduced prevalence of severe hypoglycaemia (3.5% vs. 5.9%, P = 0.02). There was no difference regarding prevalence of hypoglycaemia by dextrose dose (≤25 g vs. >25 g); however, prevalence was lower when dextrose was administered as a continuous infusion compared with bolus administration (3.3% vs. 19.5%, P = 0.02). The most common predictor of hypoglycaemia was the pre-treatment serum glucose concentration (n = 13 studies), which ranged from < 5.6–7.8 mmol/L.

**Conclusion:**

This is the first comprehensive review of the adverse effects following insulin therapy for hyperkalaemia. Hypoglycaemia remains a common adverse effect in hospitalised adults. Future randomised trials should focus on identifying the optimal regimen of insulin therapy to mitigate the risk of hypoglycaemia.

## Introduction

Hyperkalaemia is a very common electrolyte disorder encountered in hospitalized patients [1] and, if left untreated, may result in life-threatening cardiac arrhythmias and death. The emergency treatment of hyperkalaemia includes shifting potassium into cells using intravenous insulin, with dextrose added before, with or after insulin administration to prevent hypoglycaemia.

Hypoglycaemia is a frequent complication of insulin therapy, but this serious adverse effect has not yet been systematically studied and is probably under-appreciated by most clinicians. In published reports, the proportion of patients who develop hypoglycaemia is as high as 75% [2]. Hypoglycaemia has been reported to occur up to six hours following insulin treatment and therefore frequent monitoring of the serum glucose concentration is needed to detect this serious complication and prevent it from causing neurological damage [3].

Many different treatment regimens have been proposed, which have variable effects on the serum potassium concentration (K^+^) and the risk of hypoglycaemia. Insulin can either be administered as an intravenous bolus or as a continuous infusion [2, 4]. Regardless of the regimen, insulin remains the most effective non-dialytic method for reducing serum K^+^, with average reductions of approximately 1.0 mmol/L at one hour [2, 4]. One common regimen involves the intravenous bolus administration of 10 units of insulin together with 25 g of dextrose (often as 50 ml of 50% dextrose water). The insulin concentrations needed to shift potassium into cells remain above the threshold concentration only transiently; however, it remains high enough to lower blood glucose (glycaemic concentrations) for a longer period, increasing the risk of hypoglycaemia [5].

Most of the studies on which the recommended treatment regimens are based have been performed in small groups of patients with kidney failure who are being treated with chronic haemodialysis [2, 4]. Most studies used 25 g of dextrose, with the prevalence of hypoglycaemia ranging from 7% to 75% [2]. The risk for hypoglycaemia appears to increase when using smaller doses of dextrose and in patients with kidney failure. Poor kidney function increases the half-life of insulin, resulting in hypoglycaemia which typically occurs 1–3 hours after insulin administration [6, 7]. Insulins with shorter half-lives, such as lispro and aspart, may reduce the risk of hypoglycaemia in those patients with kidney disease [8]. Patients with diabetes mellitus or higher pre-treatment blood glucose concentrations have a reduced risk of hypoglycaemia [9, 10].

Given the seriousness and the apparent high frequency of hypoglycaemia following insulin therapy, and the fact that this has not been comprehensively studied, we conducted a scoping review of this important complication, and of other adverse effects of the treatment of hyperkalaemia.

### Objectives

We conducted a scoping review to map existing research on this topic and to identify any knowledge gaps. A hybrid approach was used. This included a confirmatory approach for known complications such as hypoglycaemia, as well as an exploratory approach where the included studies were screened for less well-known or unexpected complications.

The following research questions were investigated:

1. What are the reported adverse effects of insulin therapy during the emergency treatment of hyperkalaemia?
2. What is the prevalence of hypoglycaemia?
3. Have there been any other adverse effects reported?
4. What is the timing of adverse effects following therapy?
5. Have studies pre-specified surveillance for adverse effects or was the detection and reporting of adverse effects opportunistic?
6. What are the factors associated with a higher or lower risk of hypoglycaemia? We focused on the doses of insulin and dextrose used, the sequence of administration, the presence of diabetes, and pre-treatment glucose concentrations.

## Materials and methods

### Protocol and registration

We followed the *P*referred *R*eporting *I*tems for *S*ystematic *R*eviews and *M*eta-analysis–*Sc*oping *R*eviews (PRISMA-ScR) (S1 Checklist). The final protocol was registered prospectively with the Open Science Framework in September 2021 (https://osf.io/x8cs9/).

### Eligibility criteria

Research articles were eligible for inclusion if they reported any adverse effects, particularly hypoglycaemia, in adult patients (at least 18 years old), with hyperkalaemia receiving treatment that included insulin therapy. We included randomised controlled trials, prospective and retrospective cohort studies, prospective experimental cross-over studies, case-control studies, cross-sectional studies, and case series. Existing systematic reviews were also included. We excluded studies in paediatric populations, patients not treated in hospital, animal studies and case reports (S1 Table).

### Information sources

To identify relevant research, we searched the following bibliographic databases: Medline (PubMed), Embase (Ovid), the Cochrane Library (Central Register of Controlled Trials and the Cochrane Database of Systematic Reviews), CINAHL (EBSCOhost), Africa-Wide Information (EBSCOhost), Web of Science Core Collection (specifically Science Citation Index Expanded, Social Sciences Citation Index, Conference Proceedings Citation Index (Clarivate)), LILACS (Virtual Health Library) and Epistemonikos. The search strategy was tailored to each database, which is available in supporting information. All eligible research reports from 1980 to 12 October 2021, regardless of language, were included. The search strategy was performed by one of the authors (AS), an information specialist at Stellenbosch University. We also searched the conference proceedings of major nephrology congresses, specifically the American Society of Nephrology’s Kidney Week, the International Society of Nephrology’s World Congress of Nephrology and the European Renal Association-European Dialysis and Transplant Association congress, during the prior 3 years. The reference lists of retrieved publications were also hand-searched for additional relevant articles. A comprehensive search strategy can be found in S2 Table.

### Selection of eligible research

The deduplicated search yield was imported into Rayyan screening software (https://rayyan.ai/). Two reviewers (MYC and TH) independently screened all the identified articles’ titles and abstracts. Conflicts were resolved by means of discussion and reaching consensus. For those selected to be potentially eligible, we obtained the full-text articles, and these were screened by a single reviewer (MYC). Reasons were provided where studies were excluded during the full-text screening stage (S3 Table).

### Data extraction tool and data items

The first reviewer (MYC) developed and pilot-tested the data extraction tool with input from the other authors. The data that we extracted included the study design, type and year of publication, setting, sample size, demographics, comorbid and medication data, definitions of hyperkalaemia and hypoglycaemia, pre-treatment potassium and glucose concentrations, insulin regimen (type, dose, timing of administration and bolus vs. continuous infusion), dextrose administration where applicable (dose and bolus vs. continuous infusion), adverse events (timing, frequency, prespecified vs. opportunistic) and the utilisation of any other hyperkalaemia-specific therapies.

### Critical appraisal of individual sources of evidence

The AMSTAR (Assessing the Methodological Quality of Systematic Reviews) 2 tool was used to grade the quality of included systematic reviews as either high, moderate, low, or critically low [11]. This was performed by two reviewers (MYC and AS) in a non-blinded manner.

### Data synthesis

We provided a narrative synthesis of the extracted data. This was displayed in table format (S4 Table) and discussions were used to report similarities and differences between studies, as well as identifying gaps in the current evidence base.

## Results

### Selection of included research

**Fig 1:**
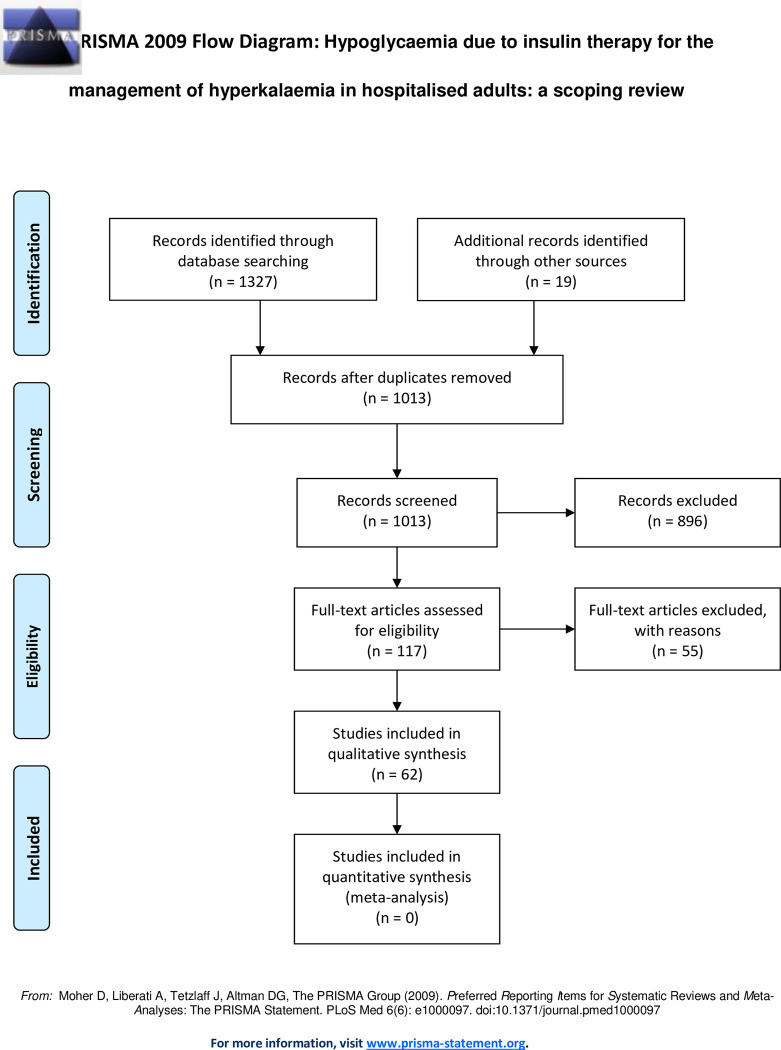
Flow diagram of study selection.

The results of the search strategy and study selection are shown in Fig 1. We included a total of 62 research articles in the scoping review [2, 4, 9, 10, 12–69].

### Characteristics of included research

Descriptive results of the primary studies are provided in Table 1. The most common study design was a retrospective cohort study (n = 38 studies); most studies were performed in North America (n = 38 studies); three quarters (n = 48 studies) were conducted from 2015 to 2021, most of which were published articles (n = 45 studies); and the most frequent definitions used for hyperkalaemia and hypoglycaemia were K+ more than 5.0 mmol/L (n = 14 studies) and a glucose concentration less than or equal to 3.9 mmol/L (n = 40 studies), respectively. Table 2 provides a summary of the individual study characteristics. The complete data set can be found as supporting information as S7 Table.

**Table 1:** Summary of characteristics of included studies.

Items	Summary
**Study design**	N = 62
Retrospective cohort	38
Prospective cohort	9
Systematic review	5
Randomised control trial	3
Prospective experimental crossover	2
Case series	1
Case-control	1
Not defined	3
**Setting (continent)**	N = 62
North America	38
Europe	11
Asia	5
Africa	2
Australia	1
**Year of publication**	N = 62
1988	2
1989	1
1990	1
1993	1
1996	1
1997	1
2001	1
2002	1
2005	1
2006	1
2012	1
2014	2
2015	3
2016	4
2017	7
2018	6
2019	11
2020	10
2021	7
**Type of publication**	N = 62
Published articles	45
Conference proceedings	17
**Range of sample size in studies**	N = 15363
Minimum	5
Maximum	1307
**Definitions for hyperkalaemia**	N = 57
>5.0 mmol/L	14
≥5.1 mmol/L	5
>5.3 mmol/L	2
>5.4 mmol/L	1
≥5.5 mmol/L	6
≥6.0 mmol/L	7
>6.1 mmol/L	1
≥7.0 mmol/L	1
Not defined	19
Other^#^	1
**Definitions for hypoglycaemia**	N = 50
<2.8 mmol/L	1
<3.0 mmol/L	2
<3.3 mmol/L	2
≤3.9 mmol/L	40
Not defined	5
**Definitions for severe hypoglycaemia**	N = 25
≤2.2 mmol/L	14
≤2.8 mmol/L	11

**Table 2:** Summary of demographic, clinical and pharmacological data for primary included studies.

	Number of treatment arms, n = 82	
**Demographic and clinical data**		
Total sample size, n	81	15 363
Age (years), mean [SD]	65	59.9 [7.4]
Sex, n		
Male	56	6 462
Not reported	27	–
Male: Female ratio	56	1.3:1
Weight (kg), mean [SD]	31	78 [7.3]
BMI (kg/m^2^), mean [SD]	14	27.2 [2.7]
Comorbidities, n		
Diabetes mellitus	46	3 575
Heart failure	2	43
Kidney transplant recipients	2	111
Kidney failure	52	5 351
Acute kidney injury	18	1 003
Chronic kidney disease (non-dialysis)	20	2 781
Chronic dialysis	42	2 254
**Pharmacological data**		
**Insulin**		
Overall average dose (units), median [IQR]	62	10 [10–10]
<10 units, median [IQR]	14	5.9 [5.0–8.7]
5–10 units	5	–
≥10 units, median [IQR]	48	10 [10–10]
Type of insulin		
Regular	47	–
Short-acting	5	–
Timing		–
Before dextrose	3	–
With dextrose	26	–
After dextrose	8	–
Infusion rate		–
Bolus	27	–
Continuous	9	–
Continuous infusion rate (min), mean [SD]	9	105 [103]
**Dextrose**		
Overall average dose (g), median [IQR]	62	25 [25–40]
≤25 g, median [IQR]	40	25 [25–25]
>25 g, median [IQR]	22	50 [40–50]
Infusion rate, %		
Bolus	19	–
Continuous	15	–
Infusion rate (min), median [IQR]	15	22.5 [15–60]
**Additional potassium-lowering therapies**		
Calcium salts	17	–
Furosemide	21	–
Sodium polystyrene sulphonate	21	–
Salbutamol nebulisation	24	–
Intravenous sodium bicarbonate	19	–
Dialysis	4	–

### Characteristics and critical appraisal of included systematic reviews

We identified five systematic reviews (S5 Table) [2, 4, 67–69]. Two reviews were regarded as being of critically low quality (no registered protocol before commencement of the review could be identified or no list and justification for the exclusion of studies or did not perform a comprehensive search strategy) [2, 68], one review was of moderate quality (two non-critical domain findings) [69] and two of high quality [4, 67].

### Synthesis of results

#### Summary of patient characteristics and pharmacological data

There was a total of 82 treatment arms among the 57 included primary studies, with 23 studies having two treatment arms, one having three treatment arms and 33 having a single treatment arm. Patient characteristics are summarised in Table 2. The total sample size was 15 363 patients. The mean [SD] age was 59.9 [7.4] years; most of the patients were male (n = 6 462 patients, n = 56 treatment arms), with a male-to-female ratio of 1.3:1; and the most frequent comorbidities were kidney failure (n = 5 351 patients) and diabetes mellitus (n = 3 575 patients). Renin-angiotensin-aldosterone system inhibitor use was reported in eight studies (n = 1 539 patients) (S1 Fig). Thirteen studies reported the use of chronic diabetic medication of which insulin therapy (n = 538 patients) was the most frequent (S2 Fig).

The data for insulin and dextrose use has been summarised in Table 2. Insulin dose was reported in 62 treatment arms. The dose could not be calculated for eight treatment arms because a dose range was reported for five treatment arms and an infusion rate (in units/kg/min)—without providing weight—was reported for three treatment arms. Regular insulin was most frequently prescribed (n = 47 treatment arms) at a median dose of 10 units [IQR 10–10 units]; insulin was administered as a bolus in most studies (n = 27 treatment arms) and was co-administered with dextrose in 26 treatment arms. The dextrose dose was available for 61 treatment arms. The dose could not be calculated for three treatment arms because a range was reported for two treatment arms and an infusion rate (mg/kg/min)—without providing weight—was reported for one treatment arm. The median dextrose dose was 25 g [IQR 25–40 g] and was most frequently administered as a bolus (n = 19 treatment arms).

Additional hyperkalaemia therapies included calcium salts (n = 17 treatment arms), furosemide (n = 21 treatment arms), sodium polystyrene sulphonate (n = 21 treatment arms), salbutamol nebulisations (n = 24 treatment arms), intravenous sodium bicarbonate (n = 19 treatment arms) and dialysis (n = 4 treatment arms) (S3 Fig).

#### What were the reported adverse effects of insulin therapy during the emergency treatment of hyperkalaemia

Nearly all the primary studies reported prespecified adverse effects (n = 55) while one study reported opportunistic adverse effects [63], and another reported both prespecified and opportunistic adverse effects [21]. Hypoglycaemia was the prespecified adverse effect reported by all primary studies. The opportunistic adverse effects reported by a single study was pulmonary oedema (one patient) and burning/warm sensations in the infusion arm or over the chest when 100 mL of 50% dextrose was administered that promptly subsided once the infusion was complete (10 patients) [21].

#### What is the prevalence of hypoglycaemia

The prevalence of hypoglycaemia (by any definition) reported by the primary studies was 17.2% [95% CI 16.6–17.8%] while the prevalence of severe hypoglycaemia (by any definition) was 5.4% [95% CI 4.9–5.9%] (Table 3). Prevalence of hypoglycaemia by specific definitions have been summarised in Table 3. The prevalence of hypoglycaemia for retrospective (n = 55 treatment arms) and prospective (n = 22 treatment arms) studies were 17.3% [95% CI 16.7–17.9%] and 15.8% [95% CI 13.4–18.5%], respectively, P = 0.54. The reported prevalence of hypoglycaemia in the systematic reviews ranged from 16.8% to 20.9% (S5 Table).

**Table 3:** Prevalence of hypoglycaemia and severe hypoglycaemia (by any definition and specified definitions).

Prevalence of hypoglycaemia	% [95% CI]
**By any definition**	17.2 [16.6–17.8]
**By specific definitions**	
<2.8 mmol/L	8.1 [5.7–11.1]
<3.0 mmol/L	8.6 [6.2–11.6]
<3.3 mmol/L	10.0 [7.9–12.5]
≤3.9 mmol/L	16.9 [16.3–17.5]
**Severe hypoglycaemia**	
** By any definition**	5.4 [4.9–5.9]
** By specific definitions**	
≤2.2 mmol/L	3.8 [3.2–4.4]
≤2.8 mmol/L	6.5 [5.8–7.3]

#### What was the timing of hypoglycaemia

Forty-three primary studies reported the duration of monitoring for hypoglycaemia, of which half monitored up to six hours following the administration of insulin therapy (S4 Fig). The median timing of hypoglycaemia occurred at 124 minutes [IQR: 102–168 minutes].

#### What were the factors associated with higher or lower risk of hypoglycaemia

There were no differences in the prevalence of hypoglycaemia when comparing insulin dose (<10 units vs. ≥10 units), rate of insulin administration (continuous vs. bolus), type of insulin (regular vs. short-acting) or timing of insulin administration relative to dextrose (before vs. with vs. after dextrose) (Table 4). However, lower insulin doses were associated with reduced prevalence of severe hypoglycaemia [3.5% vs. 5.9%, P = 0.02]. There was no difference regarding the reduction in serum K+ with lower vs. standard/high insulin doses [-0.81 mmol/L vs. -0.90 mmol/L, respectively, P = 0.18] (S5 Fig).

**Table 4:** Comparison of the prevalence of hypoglycaemia by insulin (dose, rate of infusion, type, and timing relative to dextrose) and dextrose (dose and rate of infusion).

Insulin and dextrose	Prevalence of hypoglycaemia (%), median [IQR]	p-value	Prevalence of severe hypoglycaemia (%), mean [SD]	p-value
**Insulin**				
**Dose**				
<10 units	10.9 [8.7–19.5]	0.26	3.5 [2.8]	**0.03**
≥10 units	17.5 [8.3–20.5]		5.9 [2.6]	
**Rate of infusion**				
Bolus	15.8 [6.8–22.2]	0.23	4.9 [3.4]	0.74
Continuous	6.1 [0.0–20.0]		4.0 [4.0]	
**Type**				
Regular	14.9 [8.7–20.5]	0.26	4.5 [3.0]	0.64
Short-acting	8.3 [6.1–15.8]		3.4 [3.1]	
**Timing relative to dextrose**				
Before	20.0 [2.1–75.0]	0.68	None	0.91
With	17.6 [3.3–28.6]		4.2 [2.4]	
After	13.5 [3.4–21.1]		4.0 [4.4]	
**Dextrose**				
**Dose**				
≤25g	17.1 [8.7–20.9]	0.52	5.5 [3.4]	0.19
>25g	12.5 [8.3–19.7]		4.0 [2.9]	
**Rate of infusion**				
Bolus	19.5 [11.1–27.8]	**0.02**	5.3 [3.4]	0.36
Continuous	3.3 [0.0–20.0]		3.2 [3.1]	

There was no difference regarding the prevalence of hypoglycaemia by dextrose dose (≤25 g vs. >25 g); however, prevalence was lower when dextrose was administered as a continuous infusion compared with bolus administration [3.3% vs. 19.5%, P = 0.02] (Table 4).

For comorbidities, the prevalence of hypoglycaemia was lower in treatment arms that reported this outcome in the sub-population of patients with diabetes (n = 12 treatment arms) compared to those without (n = 8 treatment arms), although this did not reach statistical significance [11.2% vs. 20.0%, P = 0.46] (S6 Fig). Also, the prevalence of hypoglycaemia was higher in treatment arms that reported this outcome in the sub-population of patients with kidney failure (n = 34 treatment arms) but was not statistically significant [19.6% vs. 10.2%, P = 0.70]. The prevalence was 22.7% for kidney transplant recipients.

Studies reporting additional hyperkalaemia therapies had lower prevalence of hypoglycaemia [14.3% vs. 18.3%, P = 0.03] (S7 Fig) as well as greater reductions in K+ [-1.0 mmol/L vs. -0.83 mmol/L, P = 0.03] when compared to studies where no additional therapies were used (S8 Fig).

Sixteen primary studies performed regression analysis [10, 12, 15, 23, 26, 29, 32, 34, 37, 46, 49, 53, 57, 59, 62, 65]. The most common predictors of hypoglycaemia were pre-treatment serum glucose concentration (n = 13 studies), insulin dose (n = 8 studies), kidney failure (n = 5 studies) and diabetes (n = 4 studies). Other predictors included weight/BMI, age, sex, and treatment in the emergency department (S6 Table).

One systematic review that we judged to be of critically low quality, performed meta-analyses and reported that alternative insulin dosing (defined as < 10 units) had lower odds associated with hypoglycaemia and severe hypoglycaemia [68].

## Discussion

This scoping review is the first comprehensive scoping review of hypoglycaemia and other adverse effects following the emergency management of hyperkalaemia. We identified 62 research articles, with most reporting on hypoglycaemia as a prespecified adverse effect and most performed during the past six years. The overall prevalence of hypoglycaemia for primary studies by any definition was 17.2% and ranged from 0% to 75%. This wide range may be due to differences in the definitions for hypoglycaemia, differences in the study populations and the insulin/dextrose regimens. For systematic reviews, the overall prevalence of hypoglycaemia had a narrow range (16.8% to 20.9%). Variation in the size of the total number of patients of included primary studies may have impacted on the observed prevalence with larger studies reporting the lowest prevalence. Another factor was the difference in the duration of monitoring for hypoglycaemia following insulin therapy which ranged from 60 minutes to 480 minutes. A study that only monitored for 60 minutes following insulin reported no episodes of hypoglycaemia [67]. However, since most systematic reviews only included prospective studies that actively monitored for hypoglycaemia, it is unlikely that episodes of hypoglycaemia were missed.

Hypoglycaemia occurred at an average of two hours following insulin therapy. It is recommended that patients with hyperkalaemia treated with insulin therapy should be monitored for up to six hours following therapy since the glucose lowering effect of insulin is prolonged in patients with kidney failure [3]. This finding is especially important since treatment is often administered in busy emergency departments where close monitoring for hypoglycaemia may be challenging.

The dose, type, rate of infusion and administration of insulin relative to dextrose were not associated with hypoglycaemia in our scoping review. However, a systematic review with meta-analysis that we judged to be of critically low quality reported a reduced prevalence of hypoglycaemia when lower doses of insulin were compared with standard doses [68]. Since there was no difference in efficacy regarding the reduction of the serum K+ with lower doses of insulin, and lower doses having a reduced risk of severe hypoglycaemia, it seems that lower doses of insulin (5 units or 0.1 units/kg) should be considered for the emergency management of hyperkalaemia.

Although a higher dextrose dose was not associated with a lower prevalence of hypoglycaemia, continuous infusion was. A theoretical regimen suggested that 60 g of 10% dextrose be infused over one hour [5]. This would require a volume of 600 mL. However, pulmonary oedema was described in a patient with kidney failure and hypertensive heart disease following 100 mL of 50% dextrose administered as a bolus [21]. Since hyperkalaemia frequently occurs in patients with kidney failure [1], decisions regarding the rate and volume of dextrose utilised should include the assessment of the patient’s volume and cardiac status.

We found that primary studies which included the use of additional hyperkalaemia therapies had a lower prevalence of hypoglycaemia. The reasons for this were unclear. There were no differences between studies that did and did not report additional therapies regarding pre-treatment serum K+, pre-treatment serum glucose concentrations, type, doses, or rate of infusion of insulin and dextrose, diabetes status and kidney failure. However, in systematic reviews, one study reported less episodes of hypoglycaemia when salbutamol nebulisations were added to insulin therapy [14], and another reported no episodes when variable combinations of salbutamol and sodium bicarbonate were added to insulin [51]. Sympathetic stimulation by salbutamol may stimulate gluconeogenesis counteracting the hypoglycaemic effect of insulin.

The most common predictor of hypoglycaemia was a lower pre-treatment serum glucose concentration. This may explain why some primary studies identified diabetes status as having a reduced risk of hypoglycaemia [15, 23, 46, 49]. Other predictors included insulin dose and kidney failure. Although we found higher prevalence rates of hypoglycaemia in patients with kidney failure and lower rates in those with diabetes, they were not statistically significant; however, the total number of treatment arms included suggests that these analyses were likely underpowered. Therefore, awareness of the pre-treatment serum glucose concentration and kidney function may be important prior to prescribing insulin therapy as these factors may help to inform the prescription so that the risk of hypoglycaemia can be reduced.

### Limitations

This scoping review has some limitations. Included studies with small sample sizes may have under- or overestimated hypoglycaemia prevalence. A few studies monitored for hypoglycaemia only up to 60 minutes. These studies may have underreported the prevalence of hypoglycaemia since this complication frequently occurs more than one hour following insulin therapy. Most of the studies were retrospective in design and hypoglycaemia was the only prespecified adverse effect. Therefore, there may be underreporting of other opportunistic adverse effects. The accuracy of some of the serum glucose measurements at the defined threshold values may have affected the prevalence of hypoglycaemia, especially when bedside capillary blood glucose measurements were performed [70]. Insulin and dextrose doses were not explicitly reported by some primary studies.

## Conclusions

This is the first comprehensive scoping review of the frequency of adverse effects following therapy with insulin for the emergency management of hyperkalaemia. Hypoglycaemia, the most concerning complication of this therapy, remains a common adverse effect but may be reduced by adapting the prescription of both insulin therapy. Most of the primary studies included in this review were retrospective in design; future randomised trials should focus on identifying the optimal regimen of insulin therapy to mitigate the risk of hypoglycaemia, while still effectively reducing K+, to inform treatment guidelines and clinical practice.

## Supporting information

S1 Checklist Preferred Reporting Items for Systematic reviews and Meta-Analyses extension for Scoping Reviews (PRISMA-ScR) checklist. (PDF)

S1 Table Inclusion and exclusion criteria. (PDF)

S2 Table Search strategy of databases. (PDF)

S3 Table List of excluded studies. (PDF)

S4 Table Individual primary study characteristics. *Kidney failure includes acute kidney injury, chronic kidney disease and end-stage kidney disease. †RCS, retrospective cohort study; ‡PCOS, Prospective cross-over study; §PCS, prospective cohort study; ‖RCT, Randomised control trial; ¶CC, case-control study; #CS, Case series; **K, Potassium.
(PDF)

S5 Table Included systematic reviews. (PDF)

S6 Table Predictors of hypoglycaemia by primary studies that performed regression analysis. All predictors included were statistically significant, P<0.05.
(PDF)

S7 Table Data set. (XLS)

S1 Fig Number of patients prescribed drugs associated with hyperkalaemia. (TIF)

S2 Fig Number of patients prescribed chronic anti-diabetic medication. (TIF)

S3 Fig Number of studies reporting additional hyperkalaemia therapies. (TIF)

S4 Fig Duration of monitoring for hypoglycaemia. (TIF)

S5 Fig Comparison of the average reduction in serum potassium concentration with <10 units vs. ≥10 units of insulin. (TIF)

S6 Fig Rates of hypoglycaemia by diabetic status. (TIF)

S7 Fig Rates of hypoglycaemia by additional hyperkalaemia therapies. (TIF)

S8 Fig Average reduction in serum potassium concentration by additional hyperkalaemia therapies. (TIF)
