# Supplementary material for: Hypoglycaemia due to insulin therapy for the management of hyperkalaemia in hospitalised adults: A scoping review
Source: PLoS One. 2022 May 12;17(5):e0268395. doi: 10.1371/journal.pone.0268395 (PMC9097985; doi:10.1371/journal.pone.0268395)
Supplement: S1 Table — (PDF) [file pone.0268395.s002.pdf]

S2A Table. Inclusion and exclusion criteria

Criteria	Inclusion	Exclusion
**Participants**	Adults (≥ 18 years old)	Paediatric patients
**Intervention / exposure**	Insulin and glucose	
**Language**	All	
**Study designs**	Systematic reviews<br>Randomised control trials<br>Cohort studies<br>Case-control studies<br>Case series<br>Cross-sectional studies	Case reports
**Publication type**	Peer-reviewed publications<br>Relevant systematic reviews<br>Conference proceedings (limited to the last 3-years)	
**Setting**	Hospitalised	Outpatients
**Time period**	January 1980 to 12 October 2021	
**Outcomes**	Any reported adverse effects following therapy with insulin and glucose for the treatment of hyperkalaemia	
