# Supplementary material for: Hypoglycaemia due to insulin therapy for the management of hyperkalaemia in hospitalised adults: A scoping review
Source: PLoS One. 2022 May 12;17(5):e0268395. doi: 10.1371/journal.pone.0268395 (PMC9097985; doi:10.1371/journal.pone.0268395)
Supplement: S2 Table — (PDF) [file pone.0268395.s003.pdf]

**S2B Table. Search strategy of databases**

| Database | Search terms |
|---|---|
| **Medline (PubMed)** | #1 "adverse effects"[MeSH Subheading] OR "complications"[MeSH Subheading] OR "deficiency"[MeSH Subheading] OR "safe"[Title/Abstract] OR "safety"[Title/Abstract] OR "side effect"[Title/Abstract] OR "side effects"[Title/Abstract] OR "undesirable effect"[Title/Abstract] OR "undesirable effects"[Title/Abstract] OR "treatment emergent"[Title/Abstract] OR "tolerability"[Title/Abstract] OR "toxicity"[Title/Abstract] OR "ADRS"[Title/Abstract] OR ("adverse"[Title/Abstract] AND ("effect"[Title/Abstract] OR "effects"[Title/Abstract] OR "reaction"[Title/Abstract] OR "reactions"[Title/Abstract] OR "event"[Title/Abstract] OR "events"[Title/Abstract] OR "outcome"[Title/Abstract] OR "outcomes"[Title/Abstract]))<br>#2 "Hypoglycemia"[Mesh] OR "hypoglycaemia"[Title/Abstract] OR "hypoglycemia"[Title/Abstract] OR "low blood sugar"[Title/Abstract] OR "low blood glucose"[Title/Abstract]<br>#3 #1 OR #2<br>#4 “Hyperkalemia” [MeSH] OR hyperkalemia [Title/Abstract] OR hyperkalaemia [Title/Abstract] OR “hyperpotassaemia” [Title/Abstract] OR “hyperpotassemia” [Title/Abstract]<br>#5 #3 AND #4<br>#6 "Insulin"[Mesh] OR insulin [Title/Abstract]<br>#7 "Glucose"[Mesh] OR glucose [Title/Abstract] OR dextrose [Title/Abstract]<br>#8 #6 AND #7<br>#9 #5 AND #8 |
| **Embase (Ovid)** | 1. adverse drug reaction/<br>2. (complication* or deficiency or safety or toxicity or tolerability).tw.<br>3. exp drug safety/<br>4. ((adverse or undesirable or harms* or serious or toxic) adj3 (effect* or reaction* or event* or outcome*)).tw.<br>5. 1 or 2 or 3 or 4<br>6. hypoglycemia/<br>7. (hypoglycaemia or hypoglycemia).tw.<br>8. ("low blood sugar" or "low blood glucose").mp. [mp=title, abstract, heading word, drug trade name, original title, device manufacturer, drug manufacturer, device trade name, keyword heading word, floating subheading word, candidate term word]<br>9. 5 or 6 or 7 or 8<br>10. hyperkalemia/<br>11. (hyperkalemia or hyperkalaemia).tw.<br>12. hyperpotassaemia.mp.<br>13. hyperpotassemia.mp.<br>14. 10 or 11 or 12 or 13<br>15. 9 and 14<br>16. insulin/ or insulin.mp.<br>17. glucose/ or glucose.mp.<br>18. dextrose.mp.<br>19. 17 or 18<br>20. 16 and 19<br>21. 15 and 20 |
| **Cochrane Library (CENTRAL AND CDSR)** | #1 MeSH descriptor: [Long Term Adverse Effects] explode all trees<br>#2 (complications):ti,ab,kw<br>#3 MeSH descriptor: [Deficiency Diseases] explode all trees<br>#4 (safe OR safety OR "side effect" OR "side effects" OR "undesirable effect" OR "undesirable effects" OR "treatment emergent" OR tolerability OR toxicity OR ADRS):ti,ab,kw<br>#5 (adverse AND effect):ti,ab,kw<br>#6 (adverse AND effects):ti,ab,kw |

| | |
|---|---|
| | #7 (adverse AND reaction):ti,ab,kw<br>#8 (adverse AND reactions):ti,ab,kw<br>#9 (adverse AND event):ti,ab,kw<br>#10 (adverse AND events):ti,ab,kw<br>#11 (adverse AND outcome):ti,ab,kw<br>#12 (adverse AND outcomes):ti,ab,kw<br>#13 MeSH descriptor: [Hypoglycemia] explode all trees<br>#14 (hypoglycaemia OR hypoglycemia OR "low blood sugar" OR "low blood glucose"):ti,ab,kw<br>#15 #1 OR #2 OR #3 OR #4 OR #5 OR #6 OR #7 OR #8 OR #9 OR #10 OR #11 OR #12 OR #13 OR #14<br>#16 MeSH descriptor: [Hyperkalemia] explode all trees<br>#17 (hyperkalemia OR hyperkalaemia OR hyperpotassaemia OR hyperpotassemia):ti,ab,kw<br>#18 #16 OR #17<br>#19 MeSH descriptor: [Insulins] explode all trees<br>#20 (insulin):ti,ab,kw<br>#21 #19 OR #20<br>#22 MeSH descriptor: [Glucose] explode all trees<br>#23 (glucose):ti,ab,kw<br>#24 (dextrose):ti,ab,kw<br>#25 #22 OR #23 OR #24<br>#26 #21 AND #25<br>#27 #15 AND #18 AND #26 |
| **Africa-wide information (EBSCOHost)** | S23. S15 AND S17 AND S22<br>S22. S19 AND S21<br>S21. TI (glucose or dextrose) OR AB (glucose or dextrose)<br>S20. SM glucose<br>S19. TI insulin OR AB insulin<br>S18. SM insulin<br>S17. TI (hyperkalemia OR hyperkalaemia OR hyperpotassaemia OR hyperpotassemia) OR AB (hyperkalemia OR hyperkalaemia OR hyperpotassaemia OR hyperpotassemia)<br>S16. SM hyperkalemia<br>S15. S4 OR S5 OR S6 OR S7 OR S8 OR S9 OR S10 OR S11 OR S12 OR S14<br>S14. TI (hypoglycaemia OR hypoglycemia OR "low blood sugar" OR "low blood glucose") OR AB (hypoglycaemia OR hypoglycemia OR "low blood sugar" OR "low blood glucose")<br>S13. SM hypoglycemia<br>S12. TI (adverse AND outcomes) OR AB (adverse AND outcomes)<br>S11. TI (adverse AND outcome) OR AB (adverse AND outcome)<br>S10. TI (adverse AND events) OR AB (adverse AND events)<br>S9. TI (adverse AND event) OR AB (adverse AND event)<br>S8. TI (adverse AND reactions) OR AB (adverse AND reactions)<br>S7. TI (adverse AND reaction) OR AB (adverse AND reaction)<br>S6. TI (adverse AND effects) OR AB (adverse AND effects)<br>S5. TI (adverse AND effect) OR AB (adverse AND effect)<br>S4. TI (safe OR safety OR "side effect" OR "side effects" OR "undesirable effect" OR "undesirable effects" OR "treatment emergent" OR tolerability OR toxicity OR ADRS) OR AB (safe OR safety OR "side effect" OR "side effects" OR "undesirable effect" OR "undesirable effects" OR "treatment emergent" OR tolerability OR toxicity OR ADRS)<br>S3. SM deficiency<br>S2. SM complications<br>S1. SM "adverse effects" |
| **Web of Science core collection (Science** | #24 AND #15 AND #18<br>#21 OR #22<br>#19 OR #20<br>glucose or dextrose (Title) or glucose or dextrose (Abstract) |

| | |
|---|---|
| **Citation Index Expanded, Social Science Citation Index, Conference Proceedings Citation Index [Clarivate])** | glucose (Topic)<br>insulin (Title) or insulin (Abstract)<br>insulin (Topic)<br>#16 OR #17<br>hyperkalemia OR hyperkalaemia OR hyperpotassaemia OR hyperpotassemia (Title) or hyperkalemia OR hyperkalaemia OR hyperpotassaemia OR hyperpotassemia (Abstract)<br>TS=(hyperkalemia)<br>#1 OR #2 OR #3 OR #4 OR #5 OR #6 OR #7 OR #8 OR #9 OR #10 OR #11 OR #12 OR #13 OR #14<br>hypoglycaemia OR hypoglycemia OR "low blood sugar" OR "low blood glucose" (Title) or hypoglycaemia OR hypoglycemia OR "low blood sugar" OR "low blood glucose" (Abstract)<br>hypoglycemia (Topic)<br>adverse AND outcomes (Title) or adverse AND outcome (Abstract)<br>adverse AND outcome (Title) or adverse AND outcomes (Abstract)<br>adverse AND events (Title) or adverse AND events (Abstract)<br>adverse AND event (Title) or adverse AND event (Abstract)<br>adverse AND reactions (Title) or adverse AND reactions (Abstract)<br>adverse AND reaction (Title) or adverse AND reaction (Abstract)<br>adverse AND effects (Title) or adverse AND effects (Abstract)<br>adverse AND effect (Title) or adverse AND effect (Abstract)<br>safe OR safety OR "side effect" OR "side effects" OR "undesirable effect" OR "undesirable effects" OR "treatment emergent" OR tolerability OR toxicity OR ADRS (Title) or safe OR safety OR "side effect" OR "side effects" OR "undesirable effect" OR "undesirable effects" OR "treatment emergent" OR tolerability OR toxicity OR ADRS (Abstract)<br>deficiency (Topic)<br>complications (Topic)<br>"adverse effects" (Topic) |
| **LILACS (Virtual Health Library)** | <u>Search 1</u><br>Adverse effects OR adverse events OR adverse reactions OR adverse outcomes OR hypoglycemia OR hypoglycaemia [Words] and hyperkalemia OR hyperkalaemia OR hyperpotassemia OR hyperpotassaemia [Words] and insulin [Words]<br><br><u>Search 2</u><br>Adverse effects OR adverse events OR adverse reactions OR adverse outcomes OR hypoglycemia OR hypoglycaemia [Words] and hyperkalemia OR hyperkalaemia OR hyperpotassemia OR hyperpotassaemia [Words] and glucose OR dextrose [Words] |
| **Epistemonikos** | <u>Search 1</u><br>(title:((title:(adverse effects OR adverse events OR adverse reactions OR adverse outcomes OR hypoglycemia OR hypoglycaemia) OR abstract:(adverse effects OR adverse events OR adverse reactions OR adverse outcomes OR hypoglycemia OR hypoglycaemia)) AND (title:(hyperkalemia OR hyperkalaemia OR hyperpotassemia OR hyperpotassaemia) OR abstract:(hyperkalemia OR hyperkalaemia OR hyperpotassemia OR hyperpotassaemia)) AND (title:(glucose OR dextrose) OR abstract:(glucose OR dextrose))) OR abstract:((title:(adverse effects OR adverse events OR adverse reactions OR adverse outcomes OR hypoglycemia OR hypoglycaemia) OR abstract:(adverse effects OR adverse events OR adverse reactions OR adverse outcomes OR hypoglycemia OR hypoglycaemia)) AND (title:(hyperkalemia OR hyperkalaemia OR hyperpotassemia OR hyperpotassaemia) OR abstract:(hyperkalemia OR hyperkalaemia OR hyperpotassemia OR hyperpotassaemia)) AND (title:(glucose OR dextrose) OR abstract:(glucose OR dextrose))))<br><br><u>Search 2</u> |

| | |
|---|---|
| | (title:((title:(adverse effects OR adverse events OR adverse reactions OR adverse outcomes OR hypoglycemia OR hypoglycaemia) OR abstract:(adverse effects OR adverse events OR adverse reactions OR adverse outcomes OR hypoglycemia OR hypoglycaemia)) AND (title:(hyperkalemia OR hyperkalaemia OR hyperpotassemia OR hyperpotassaemia) OR abstract:(hyperkalemia OR hyperkalaemia OR hyperpotassemia OR hyperpotassaemia)) AND (title:(insulin) OR abstract:(insulin))) OR abstract:((title:(adverse effects OR adverse events OR adverse reactions OR adverse outcomes OR hypoglycemia OR hypoglycaemia) OR abstract:(adverse effects OR adverse events OR adverse reactions OR adverse outcomes OR hypoglycemia OR hypoglycaemia)) AND (title:(hyperkalemia OR hyperkalaemia OR hyperpotassemia OR hyperpotassaemia) OR abstract:(hyperkalemia OR hyperkalaemia OR hyperpotassemia OR hyperpotassaemia)) AND (title:(insulin) OR abstract:(insulin)))) |
