# Supplementary material for: Hypoglycaemia due to insulin therapy for the management of hyperkalaemia in hospitalised adults: A scoping review
Source: PLoS One. 2022 May 12;17(5):e0268395. doi: 10.1371/journal.pone.0268395 (PMC9097985; doi:10.1371/journal.pone.0268395)
Supplement: S3 Table — (PDF) [file pone.0268395.s004.pdf]

S2C Table. List of excluded studies

Study	Reason for exclusion
Ahmad et al. 2010	No adverse effects reported
Al-Sharefi et al. 2019	Letter
Alfonzo et al. 2006	Review
Aljabri et al. 2017	Full text used
Allon et al. 1993	No baseline hyperkalaemia
Alsulami et al. 2019	Full text used
Boughton et al. 2019	Letter
Boughton et al. 2019	Duplicate
Broz et al. 2018	Letter
Broz et al. 2019	Letter
Centeno et al. 2017	Not a hyperkalaemia-specific study
Depret et al. 2019	Review
Doshi et al. 2016	Review
Driver et al. 2017	Multiple causes of hypoglycaemia
Effa et al. 2017	Review
Evans et al. 2004	Review
Farina et al. 2016	Full text used
Fischer et al. 1986	Multiple causes of hypoglycaemia
Goksu et al. 2003	No adverse effects reported
Groene et al. 2017	Review
Hendra et al. 2016	No adverse effects reported
Humphrey et al. 2019	Full text used
Jamal et al. 2018	Information sparse
Janjua et al. 2011	Paediatric study
Kraft et al. 2005	Review
Kraft et al. 2006	Letter
Krishnan 2002	Review
LaRue et al. 2015	Full text used
Li et al. 2014	Review
Liu et al. 2019	Review
Long et al. 2019	Letter
Macedo et al. 2017	No adverse effects reported
Mastroianni et al. 2017	Adverse effects related to insulin not clear
Maxwell et al. 2013	Review
McVeigh 2003	Review
Moussavi et al. 2019	Review
Moussavi et al. 2021	Full text used
Mustafa et al. 2014	Editorial
Palaka et al. 2017	Only resins investigated
Paparella et al. 2018	Letter
Peacock et al. 2016	Data unavailable
Peacock et al. 2020	No insulin-based therapy
Putcha et al. 2007	Review
Rajendran et al. 2014	Survey
Raymond et al. 2010	Review
Rossignol et al. 2016	Guideline
Sacchetti et al. 1999	Dialytic therapy only
Sridhar et al. 2018	Insulin for hyperglycaemia
Tabatabai et al. 2014	Survey
Tzamaloukas et al. 1987	Insulin for hyperglycaemia
Verdier et al. 2020	Full text used
Xia et al. 2010	Rates of hypoglycaemia not reported
Yorifuji et al. 2013	Multiple causes of hypoglycaemia
Zahoor et al. 2012	Hyperkalaemia post-parathyroidectomy
No author	Unidentifiable paper
