# Supplementary material for: Hypoglycaemia due to insulin therapy for the management of hyperkalaemia in hospitalised adults: A scoping review
Source: PLoS One. 2022 May 12;17(5):e0268395. doi: 10.1371/journal.pone.0268395 (PMC9097985; doi:10.1371/journal.pone.0268395)
Supplement: S4 Table — *Kidney failure includes acute kidney injury, chronic kidney disease and end-stage kidney disease. †RCS, retrospective cohort study; ‡PCOS, Prospective cross-over study; §PCS, prospective cohort study; ‖RCT, Randomised control trial; ¶CC, case-control study; #CS, Case series; **K, Potassium. (PDF) [file pone.0268395.s005.pdf]

S2D Table. Individual primary study characteristics

Author	Year	Study design	Sample	Age	Male	KF*	Definition hyperka	Definition hypogly	Duration of monitor	Pretreatment [K+]	Pretreatment [glucos	Insulin (IU)	Insulin (IU/kg)	Dextrose (g)	[K+] reduction, mM	% Hypoglycaemia
**Aljabri (12)**	2019	RCS	90	62	48	Yes	>5.0	≤3.9	6	6	7	10	–	25	N/D	22
**Aljabri (13)**	2021	RCS	521	62	271	Yes	>5.0	≤3.9	6	6	8	10	–	25	N/D	2
**Allon (14)**	1990	PCOS	10	57	N/D	Yes	>5.0	N/D	1	6	5	10	–	25	-1.21	20
**Allon (14)**	1990	PCOS	12	57	N/D	Yes	>5.0	N/D	1	6	5	10	–	25	-0.65	75
**Apel (9)**	2014	RCS	221	51	18	Yes	N/D	<3.3	6	N/D	6	10	–	25	N/D	13
**Beltrami-Moreira (15)**	2020	PCS	160	68	78	Yes	N/D	≤3.9	12	N/D	N/D	5–10	N/D	N/D	N/D	18
**Binz (16)**	2020	RCS	1291	62	752	Yes	N/D	≤3.9	N/D	6	8	–	–	25	N/D	18
**Blumberg (17)**	1988	PCS	10	58	5	Yes	N/D	N/D	N/D	6	5	–	5u/kg/min	5mg/kg/min	-0.90	50
**Boughton (18)**	2019	RCS	662	71	617	Yes	N/D	≤3.9	N/D	6	6	10	–	20	-0.60	18
**Brown (19)**	2018	RCS	264	56	168	Yes	>5.0	≤3.9	8	6	9	8	0.1	24	-0.60	7
**Brown (19)**	2018	RCS	69	57	64	Yes	>5.0	≤3.9	8	6	9	9	–	26	-0.60	16
**Chittineni (20)**	2019	RCS	61	49	N/D	N/D	N/D	≤3.9	N/D	N/D	N/D	–	–	N/D	N/D	74
**Chothia (21)**	2014	RCT	10	40	5	Yes	>5.0	<3.0	N/D	6	6	10	–	50	-0.83	20
**Coca (10)**	2017	RCS	164	72	6	Yes	≥6.0	≤3.9	8	7	6	10	–	50	-1.37	6
**Coca (22)**	2017	CC	30	76	18	Yes	N/D	N/D	4	7	7	10	–	50	-0.86	10
**Coca (22)**	2017	CC	30	76	18	Yes	N/D	N/D	4	7	8	10	–	50	-0.82	3

Author	Year	Study design	Sample	Age	Male	KF*	Definition hyperka	Definition hypogly	Duration of monitor	Pretreatment [K+]	Pretreatment [glucos	Insulin (IU)	Insulin (IU/kg)	Dextrose (g)	[K+] reduction, mM	% Hypoglycaemia
**Crnobrnja (23)**	2020	RCS	421	71	60	Yes	≥6.0	≤3.9	6	N/D	7	10	–	25	N/D	21
**Diveley (24)**	2021	RCS	90	N/D	N/D	Yes	>5.0	≤3.9	N/D	N/D	N/D	10	–	N/D	N/D	17
**Diveley (24)**	2021	RCS	49	N/D	N/D	No	>5.0	≤3.9	N/D	N/D	N/D	10	–	N/D	N/D	10
**Dixon (25)**	2016	RCS	57	71	N/D	N/D	N/D	<4.0	N/D	N/D	N/D	–	–	N/D	N/D	30
**Dixon (25)**	2016	RCS	59	69	N/D	N/D	N/D	<4.0	N/D	N/D	N/D	–	–	N/D	N/D	12
**Do (26)**	2019	RCS	1156	61	682	Yes	>5.0	≤3.9	N/D	6	7	0.1 Iu/kg	0.105	N/D	N/D	18
**Driver (27)**	2016	RCS	433	N/D	N/D	N/D	>5.3	<2.8	2	N/D	N/D	10	–	34	N/D	8
**Farina (28)**	2018	RCS	120	60	69	Yes	>5.0	≤3.9	4	7	7	10	–	25	-1.00	16
**Farina (28)**	2018	RCS	120	62	61	Yes	>5.0	≤3.9	4	6	6	10	–	50	-1.10	8
**Garcia (29)**	2018	RCS	309	59	219	Yes	≥5.1	≤3.9	6	6	9	10	–	50	-0.90	11
**Garcia (29)**	2018	RCS	92	62	63	Yes	≥5.1	≤3.9	6	6	8	5	–	19	-0.81	9
**Hain (30)**	2020	N/D	105	62	N/D	Yes	N/D	≤3.9	3	6	7	10	–	50	-1.10	14
**Humphrey (31)**	2020	RCS	110	56	N/D	Yes	≥5.5	<4.0	6	6	N/D	–	–	N/D	-0.89	23
**Humphrey (32)**	2021	RCS	1284	72	804	Yes	≥5.5	<4.0	6	6	8	10	–	25	-0.86	19
**Jacob (33)**	2019	RCS	172	63	154	Yes	≥5.1	≤3.9	24	7	5	10	0.11	25	-2.10	20
**Keeney (34)**	2019	RCS	295	60	178	Yes	N/D	≤3.9	6	7	7	10	–	2	-1.13	16

Author	Year	Study design	Sample	Age	Male	KF*	Definition hyperka	Definition hypogly	Duration of monitor	Pretreatment [K+]	Pretreatment [glucos	Insulin (IU)	Insulin (IU/kg)	Dextrose (g)	[K+] reduction, mM	% Hypoglycaemia
**Keeney (34)**	2019	RCS	147	65	76	Yes	N/D	≤3.9	6	7	7	5	–	2	-1.17	6
**Kim (35)**	1996	PCOS	8	52	6	Yes	>6.1	N/D	1	6	6	50	6 [illegible]	20	-1.10	0
**Kocoglu (36)**	2002	PCS	14	53	10	No	≥8.0	N/D	N/D	N/D	N/D	25	–	50	-0.08	0
**Kocoglu (36)**	2002	PCS	22	52	14	No	≥7.0	N/D	N/D	N/D	N/D	50	–	100	-1.78	36
**Konowitz (37)**	2019	RCS	1307	N/D	N/D	N/D	N/D	≤3.9	N/D	6	N/D	–	–	N/D	N/D	18
**LaRue (38)**	2017	RCS	542	62	272	Yes	>5.0	≤3.9	5	6	8	10	–	39	-1.00	29
**LaRue (38)**	2017	RCS	133	60	86	Yes	>5.0	≤3.9	5	6	7	5	–	34	-1.00	20
**Lane (40)**	1989	PCS	10	61	5	Yes	N/D	N/D	6	7	8	10	–	40	-1.00	20
**Lane (40)**	1989	PCS	10	62	4	Yes	N/D	N/D	6	7	7	10	–	40	-1.50	0
**Lim (41)**	2019	RCS	96	N/D	N/D	N/D	≥5.5	≤3.9	12	N/D	N/D	–	–	N/D	N/D	19
**Ljutic (42)**	1993	PCS	9	N/D	5	Yes	>5.0	<3.0	1	6	6	10	–	25	-0.76	22
**Macmaster (43)**	2017	N/D	100	N/D	N/D	N/D	≥5.1	≤3.9	6	N/D	N/D	10	–	N/D	N/D	23
**Macmaster (44)**	2018	N/D	146	N/D	N/D	N/D	≥5.1	≤3.9	6	N/D	N/D	–	–	50	N/D	10
**Macmaster (44)**	2018	N/D	225	N/D	N/D	N/D	≥5.1	≤3.9	6	N/D	N/D	10	–	25	N/D	21
**Mahajan (45)**	2001	PCS	15	N/D	N/D	N/D	≥6.0	<3.3	6	7	N/D	–	–	N/D	-0.80	7
**Mansour (46)**	2019	RCS	142	N/D	N/D	Yes	>5.4	≤3.9	6	N/D	N/D	–	–	N/D	N/D	18

Author	Year	Study design	Sample	Age	Male	KF*	Definition hyperka	Definition hypogly	Duration of monitor	Pretreatment [K+]	Pretreatment [glucos	Insulin (IU)	Insulin (IU/kg)	Dextrose (g)	[K+] reduction, mM	% Hypoglycaemia
**McNicholas (47)**	2017	RCS	63	50	41	Yes	≥6.0	≤3.9	6	7	10	5–10	–	25	N/D	11
**McNicholas (47)**	2017	RCS	76	56	53	Yes	≥6.0	≤3.9	6	7	7	5–10	–	25	-0.57/10	29
**Meloy (48)**	2021	RCS	128	N/D	N/D	Yes	≥5.5	≤3.9	6	N/D	N/D	5–10	–	2	N/D	42
**Moussavi (49)**	2020	RCS	223	60	115	Yes	>5.0	≤3.9	12	6	6	5	0.07	25	-0.94	11
**Moussavi (49)**	2020	RCS	477	62	259	Yes	>5.0	≤3.9	12	6	8	10	0.14	25	-1.11	18
**Mushtaq (50)**	2006	PCS	5	51	N/D	Yes	≥6.0	N/D	6	7	8	10	–	25	-1.10	0
**Mushtaq (50)**	2006	PCS	5	52	N/D	Yes	≥6.0	N/D	6	7	8	10	–	25	-0.80	0
**Ngugi (51)**	1997	RCT	10	N/D	N/D	Yes	>5.0	N/D	8	N/D	7	10	–	25	-0.90	0
**Ngugi (51)**	1997	RCT	10	N/D	N/D	Yes	>5.0	N/D	8	N/D	5	10	–	25	-1.39	20
**Ngugi (51)**	1997	RCT	10	N/D	N/D	Yes	>5.0	N/D	8	N/D	7	10	–	25	-1.19	0
**Peacock (52)**	2018	PCS	130	56	124	Yes	≥5.5	N/D	4	6	N/D	–	–	N/D	-1.00	6
**Pearson (53)**	2021	RCS	182	64	108	Yes	N/D	≤3.9	6	6	8	10	–	25	-0.60	19
**Pearson (53)**	2021	RCS	204	65	125	Yes	N/D	≤3.9	6	6	8	10	–	25	-0.90	18
**Pierce (54)**	2015	RCS	78	58	71	Yes	≥6.0	≤3.9	8	6	N/D	10	–	25	-1.08	17
**Pierce (54)**	2015	RCS	71	62	63	Yes	≥6.0	≤3.9	8	6	N/D	5	–	25	-1.10	20
**Rafique (55)**	2020	RCT	15	48	7	Yes	≥6.0	≤3.9	N/D	7	6	5	–	N/D	-0.60	20

Author	Year	Study design	Sample	Age	Male	KF*	Definition hyperka	Definition hypogly	Duration of monitor	Pretreatment [K+]	Pretreatment [glucos	Insulin (IU)	Insulin (IU/kg)	Dextrose (g)	[K+] reduction, mM	% Hypoglycaemia
**Schafers (56)**	2012	RCS	219	53	15	Yes	≥6.0	≤3.9	6	N/D	7	5–10	N/D	0–50	N/D	9
**Scott (57)**	2018	RCS	409	57	N/D	Yes	>5.3	≤3.9	3	6	6	10	–	40	N/D	17
**Szwak (58)**	2017	RCS	141	N/D	N/D	Yes	N/D	N/D	N/D	N/D	N/D	–	–	N/D	-0.80	26
**Tee (59)**	2020	RCS	132	63	19	Yes	(≥6.5 or [illegible]	<4.0	6	6	6	10	–	20–25	-0.77	21
**Tran (60)**	2020	PCS	225	59	147	Yes	≥5.1	≤3.9	6	6	9	10	0.12	25	-0.73	21
**Tran (60)**	2020	PCS	145	57	85	Yes	≥5.1	≤3.9	6	6	10	7	0.09	25	-0.95	10
**Verdier (61)**	2021	RCS	87	61	48	Yes	>5.0	≤3.9	6	6	8	5	–	44	-0.80	9
**Verdier (61)**	2021	RCS	87	63	52	Yes	>5.0	≤3.9	6	6	8	10	–	47	-0.70	20
**Wheeler (62)**	2016	RCS	66	56	37	Yes	N/D	≤3.9	24	6	9	10	–	50	-1.35	20
**Wheeler (62)**	2016	RCS	66	62	41	Yes	N/D	≤3.9	24	6	8	7	0.1	50	-1.34	11
**Williams (63)**	1988	CS	N/D	39	2	Yes	N/D	N/D	N/D	7	N/D	14	–	20	N/D	N/D
**Wu (64)**	2015	RCS	191	61	16	Yes	N/D	≤3.9	6	N/D	8	10	–	25	N/D	14
**Yang (65)**	2019	RCS	62	67	40	Yes	≥5.5	≤3.9	24	6	6	10	0.12	50	-0.80	26
**Yang (65)**	2019	RCS	72	71	46	Yes	≥5.5	≤3.9	24	6	7	10	0.12	25	-0.90	22
**Zuern (66)**	2020	RCS	90	59	62	Yes	>5.0	≤3.9	4	6	7	10	0.11	25	-0.80	28
**Zuern (66)**	2020	RCS	75	61	44	Yes	>5.0	≤3.9	4	6	7	9	0.1	25	-0.60	13

*Kidney failure includes acute kidney injury, chronic kidney disease and end-stage kidney disease. †RCS, retrospective cohort study; ‡PCOS, Prospective cross-over study; §PCS, prospective cohort study; ‖RCT, Randomised control trial; ¶CC, case-control study; #CS, Case series; **K, Potassium.
