# Supplementary material for: Hypoglycaemia due to insulin therapy for the management of hyperkalaemia in hospitalised adults: A scoping review
Source: PLoS One. 2022 May 12;17(5):e0268395. doi: 10.1371/journal.pone.0268395 (PMC9097985; doi:10.1371/journal.pone.0268395)
Supplement: S5 Table — (PDF) [file pone.0268395.s006.pdf]

S2E Table. Included systematic reviews

Author name	Year of publication	Aim	Eligibility criteria	No. of studies that included insulin	Reported adverse events	Quality (AMSTAR)
Batterink et al. (67)	2015	Benefits and harms of pharmacological treatments used in the acute management of hyperkalaemia in adults and evaluated the therapies that reduce serum potassium as well as those that prevent complications of hyperkalaemia.	Adults (aged 18 years and over) with hyperkalaemia, defined as serum potassium concentration >4.9 mmol/L, to receive pharmacological therapy to reduce serum potassium or to prevent arrhythmias.	3	14 of 67 patients with hypoglycaemia (20.9%)	High
Harel et al. (2)	2016	Review data in the literature to determine the optimal dose and route of administration of insulin in the management of emergency hyperkalaemia.	Articles were eligible for inclusion if they reported on using insulin in the management	11	22 of 117 patients with hypoglycaemia (18.8%)	Critically low
Varallo et al. (70)	2019	To evaluate the efficacy, effectiveness, and safety of hyperkalaemia pharmacotherapies.	The population included patients with hyperkalaemia (without restrictions for age, sex, or current or previous past medical history) receiving hyperkalaemia treatment compared with placebo, no treatment, or another comparator.	5	Two patients with hypoglycaemia), One patient with pulmonary oedema)	Moderate

Author name	Year of publication	Aim	Eligibility criteria	No. of studies that included insulin	Reported adverse events	Quality (AMSTAR)
Mahoney et al. (68)	2005	To review randomised evidence informing the acute emergency management of hyperkalaemia.	Studies reporting pharmacological or other interventions to treat non-neonatal humans with hyperkalaemia, reporting on clinically important outcomes, or serum potassium levels within the first six hours of administration.	4	14 of 83 patients with hypoglycaemia (16.9%)	High
Moussavi et al. (69)	2021	To determine the impact of alternative insulin dosing on hypoglycaemia and potassium reduction in patients with hyperkalaemia.	Patients treated with standard (10 units) or alternative (<10 units) insulin	10	Low dose insulin: 124 of 1084 patients with hypoglycaemia (11.4%); Standard dose insulin: 452 of 2353 patients with hypoglycaemia (19.2%); Total 576 of 3437 patients with hypoglycaemia (16.8%)	Critically low
