# Supplementary material for: Hypoglycaemia due to insulin therapy for the management of hyperkalaemia in hospitalised adults: A scoping review
Source: PLoS One. 2022 May 12;17(5):e0268395. doi: 10.1371/journal.pone.0268395 (PMC9097985; doi:10.1371/journal.pone.0268395)
Supplement: S6 Table — All predictors included were statistically significant, P<0.05. (PDF) [file pone.0268395.s007.pdf]

S2F Table. Predictors of hypoglycaemia by primary studies that performed regression analysis

Predictors of hypoglycaemia	Number of studies
Pre-treatment blood glucose	13
Insulin dose	8
Kidney failure	5
Diabetes	4
Weight/Body mass index	2
Age	2
Male sex	1
Female sex	1
Emergency department treatment	2

All predictors included were statistically significant, $P<0.05$
