# Supplementary figures and images for: Hypoglycaemia due to insulin therapy for the management of hyperkalaemia in hospitalised adults: A scoping review
Source: PLoS One. 2022 May 12;17(5):e0268395. doi: 10.1371/journal.pone.0268395 (PMC9097985; doi:10.1371/journal.pone.0268395)

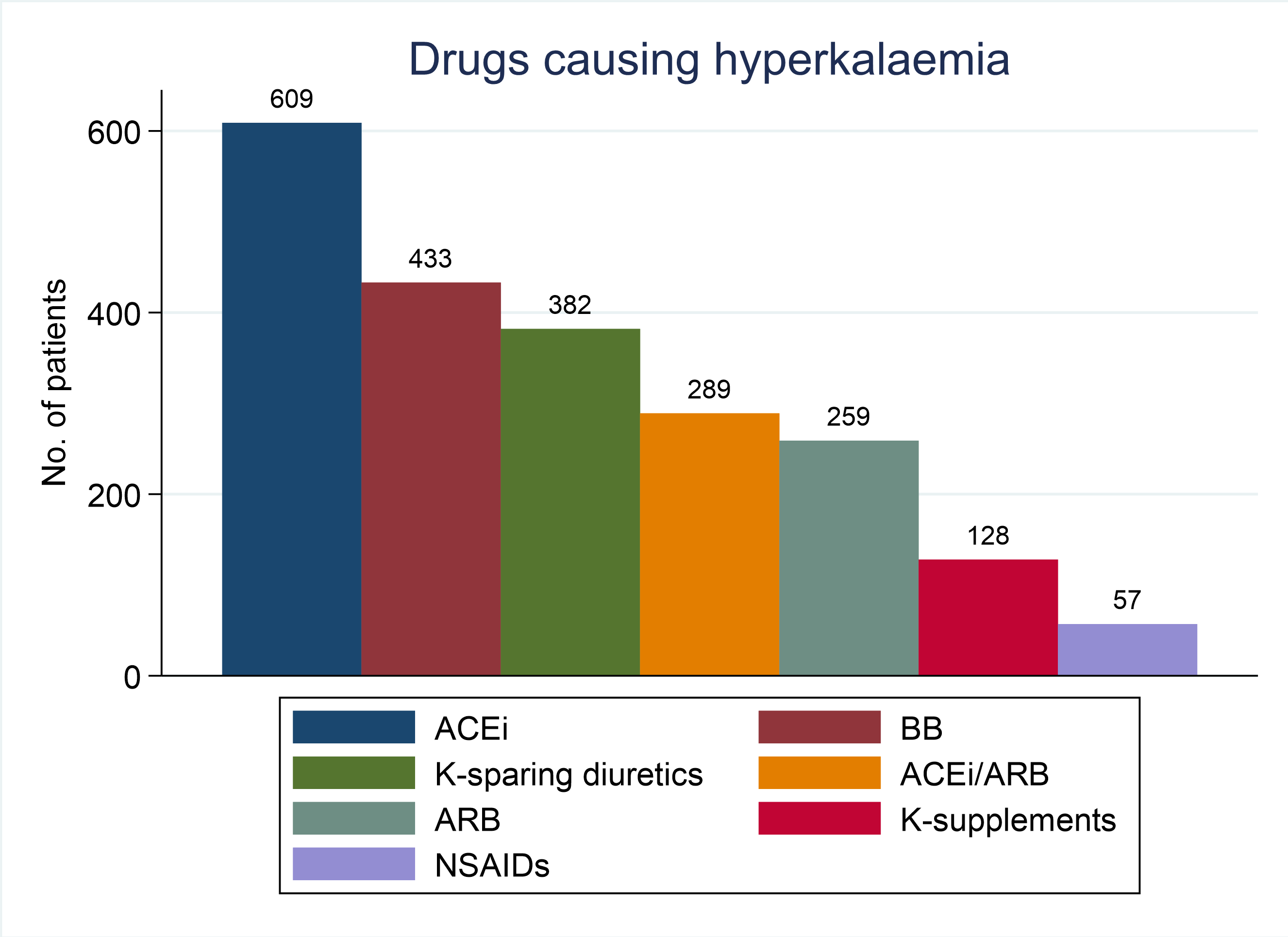

Supplement: S1 Fig — (TIF) [file pone.0268395.s009.tif]

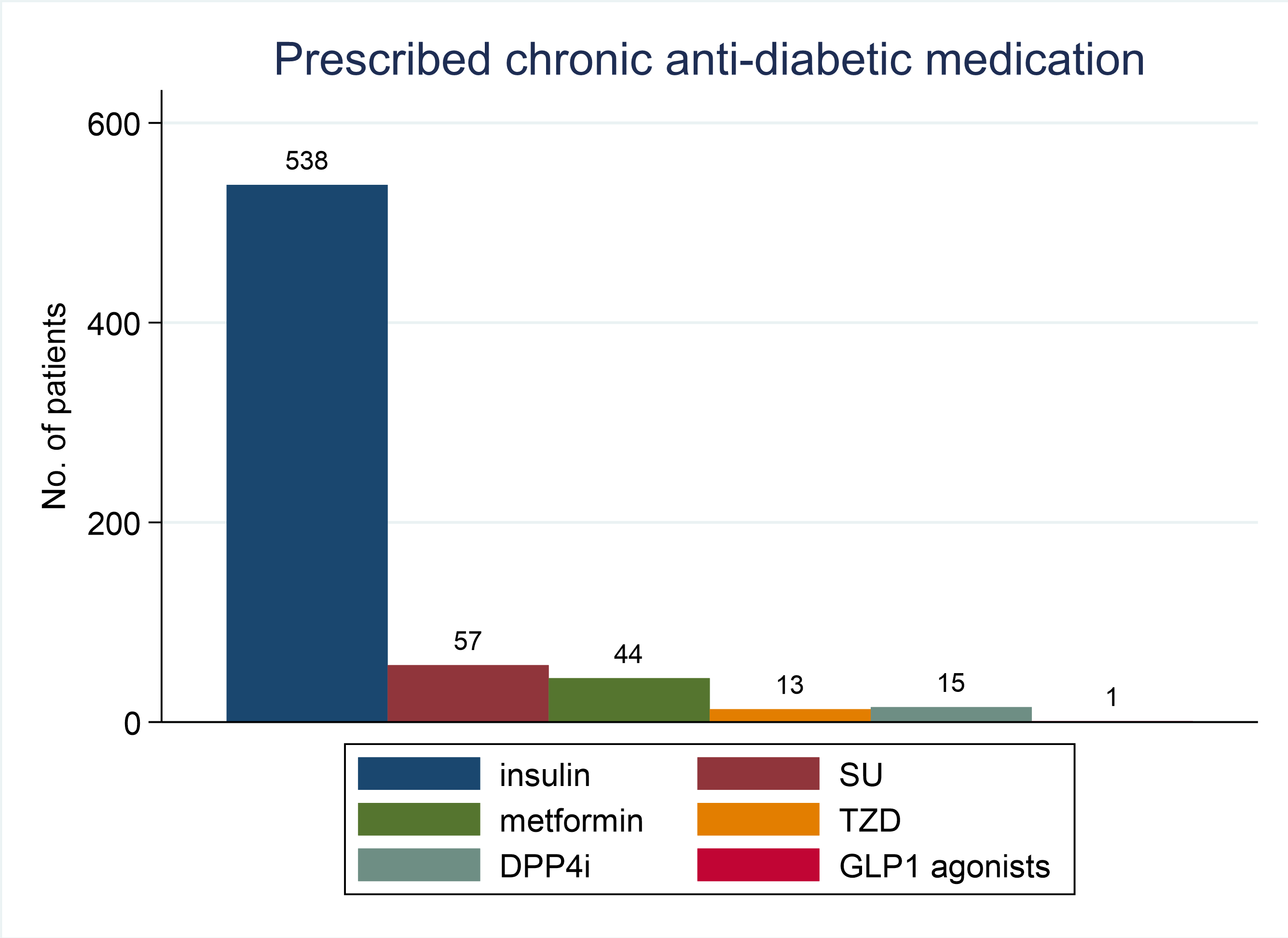

Supplement: S2 Fig — (TIF) [file pone.0268395.s010.tif]

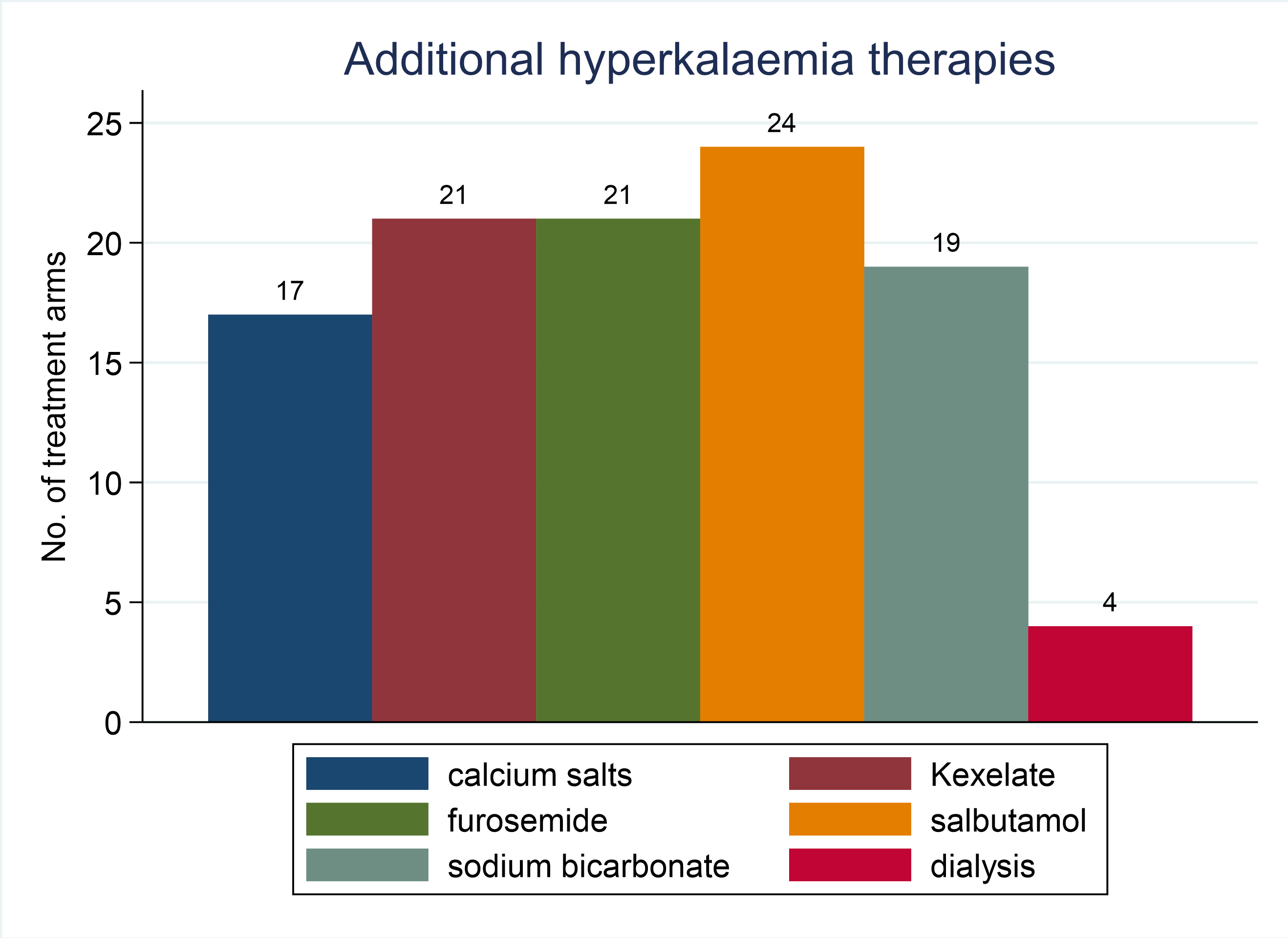

Supplement: S3 Fig — (TIF) [file pone.0268395.s011.tif]

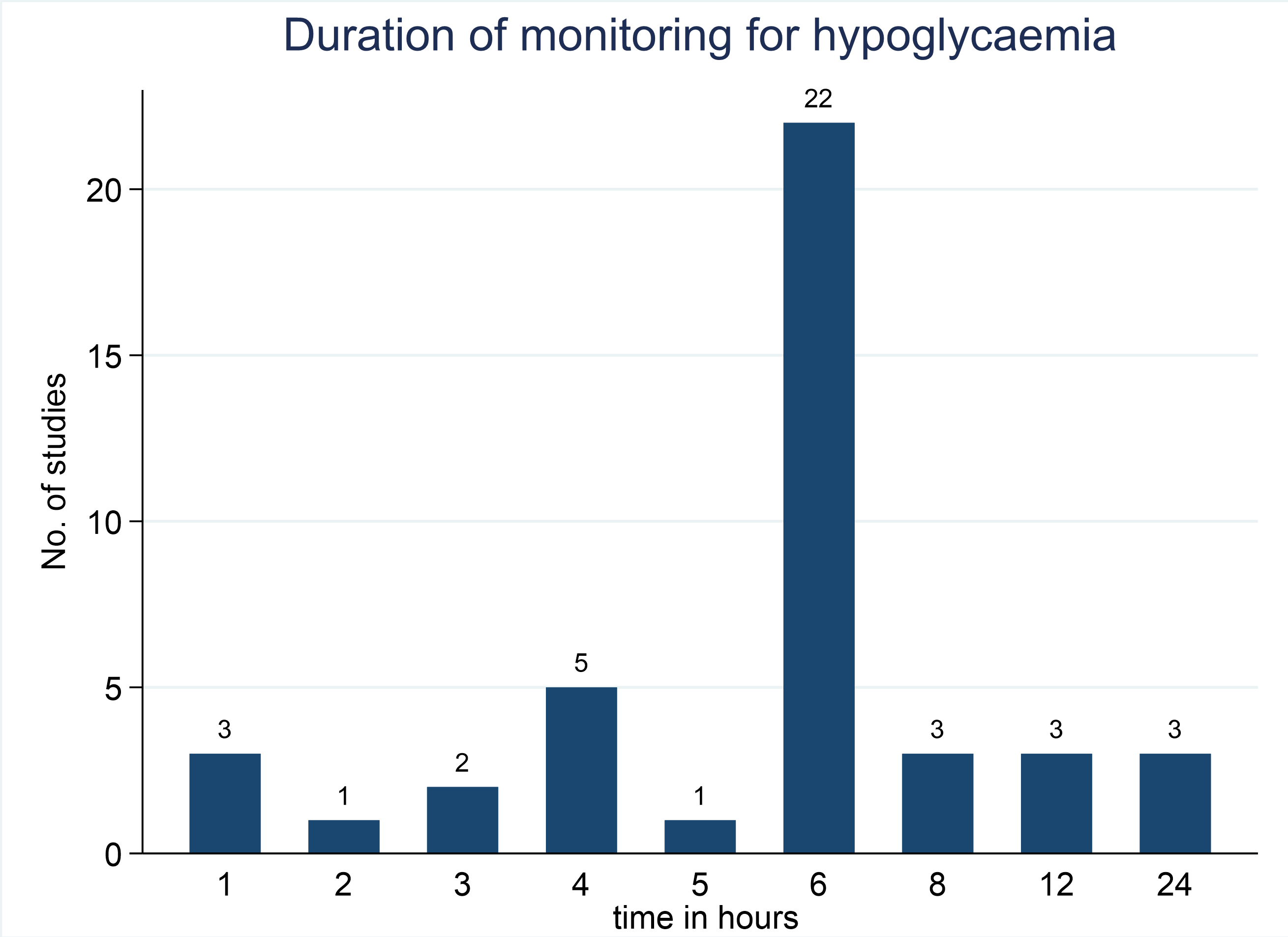

Supplement: S4 Fig — (TIF) [file pone.0268395.s012.tif]

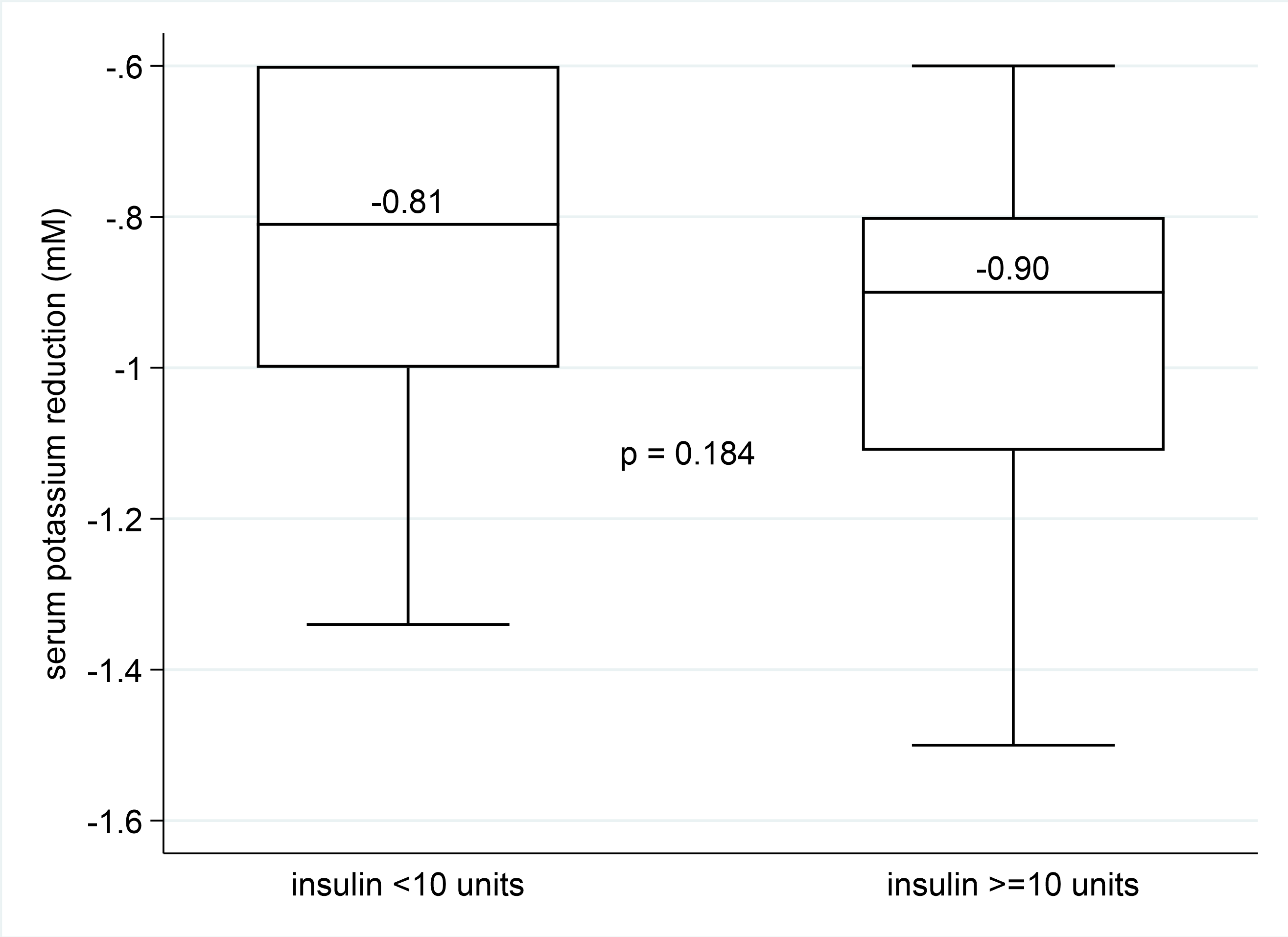

Supplement: S5 Fig — (TIF) [file pone.0268395.s013.tif]

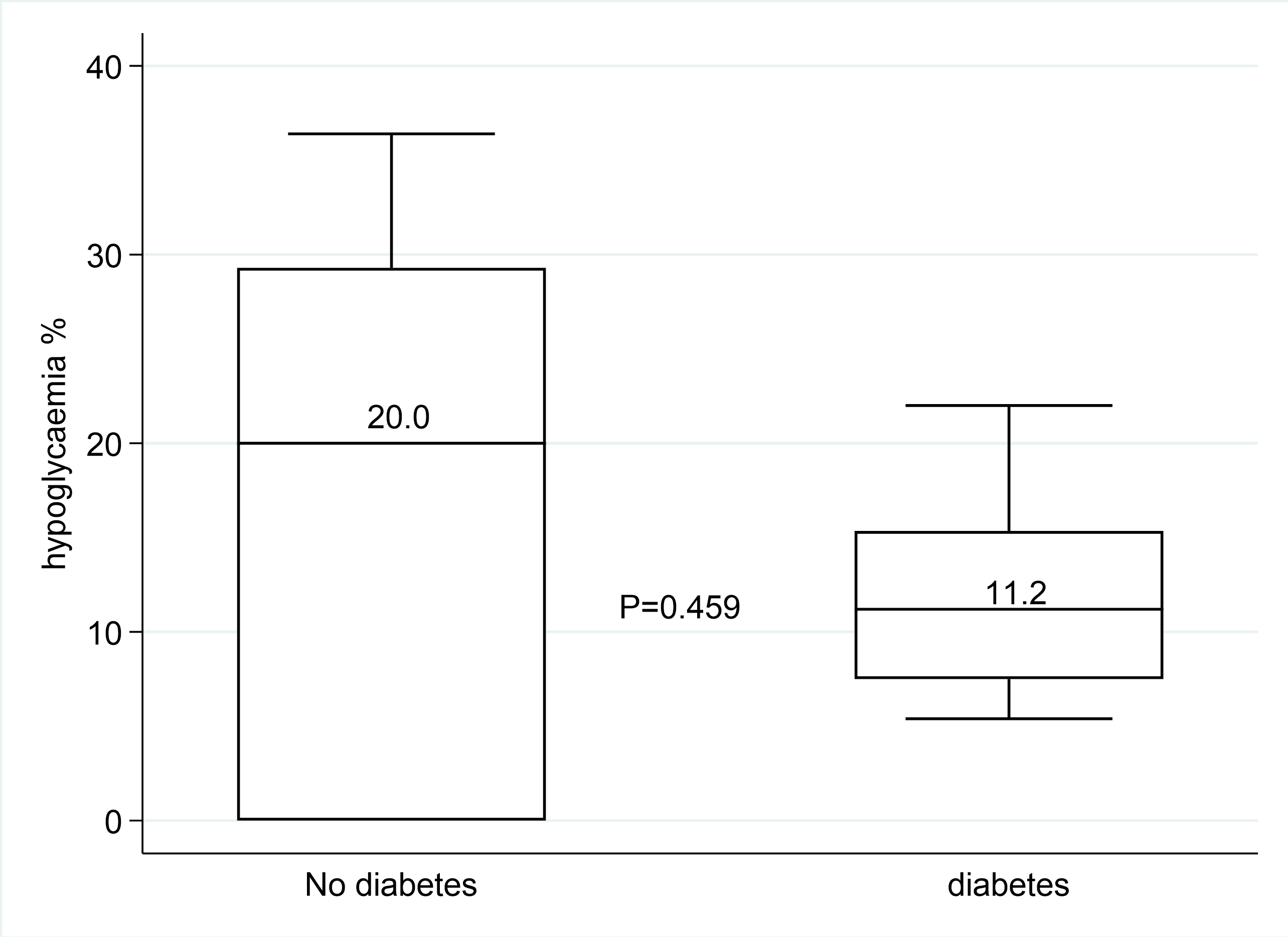

Supplement: S6 Fig — (TIF) [file pone.0268395.s014.tif]

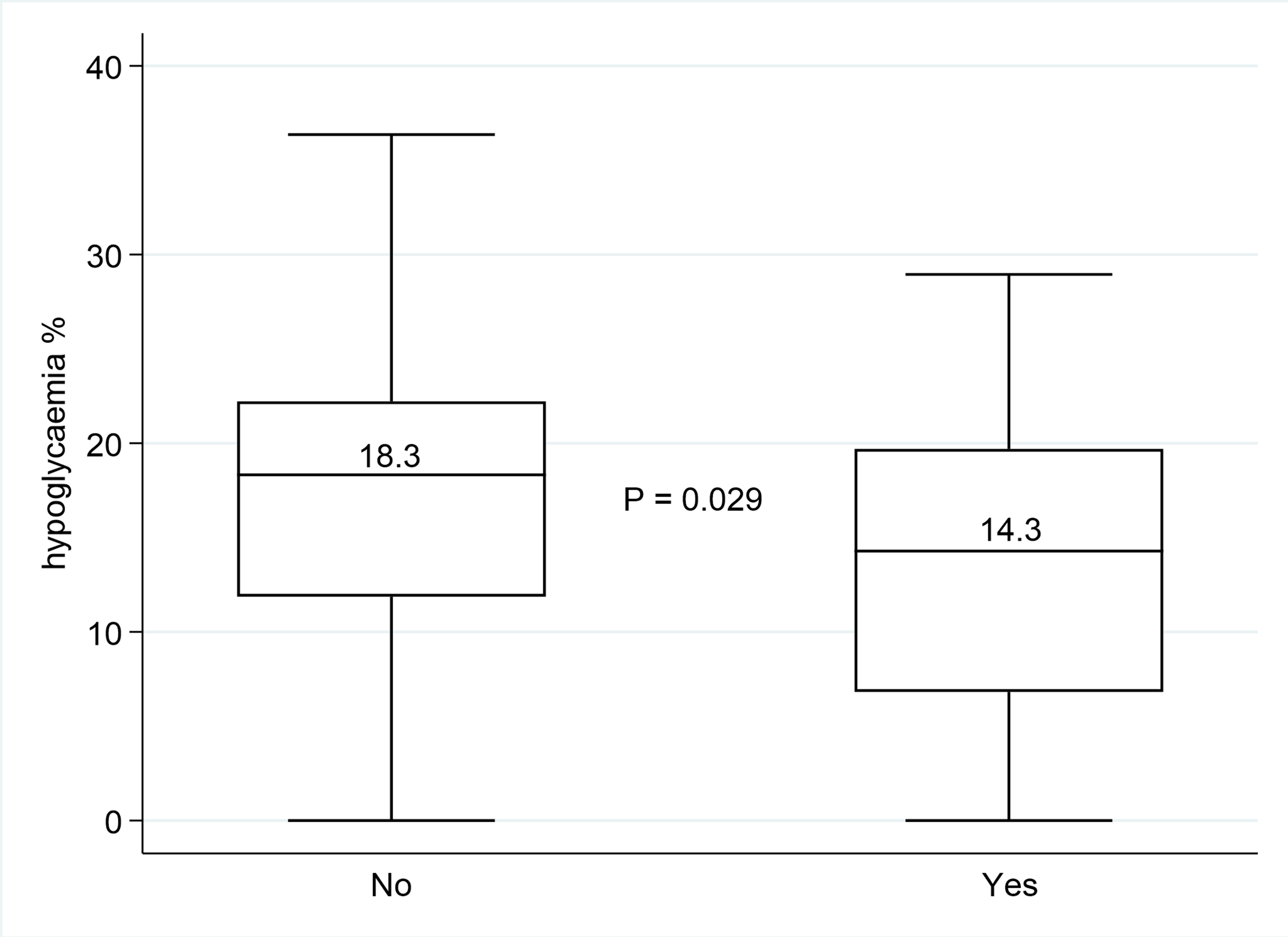

Supplement: S7 Fig — (TIF) [file pone.0268395.s015.tif]

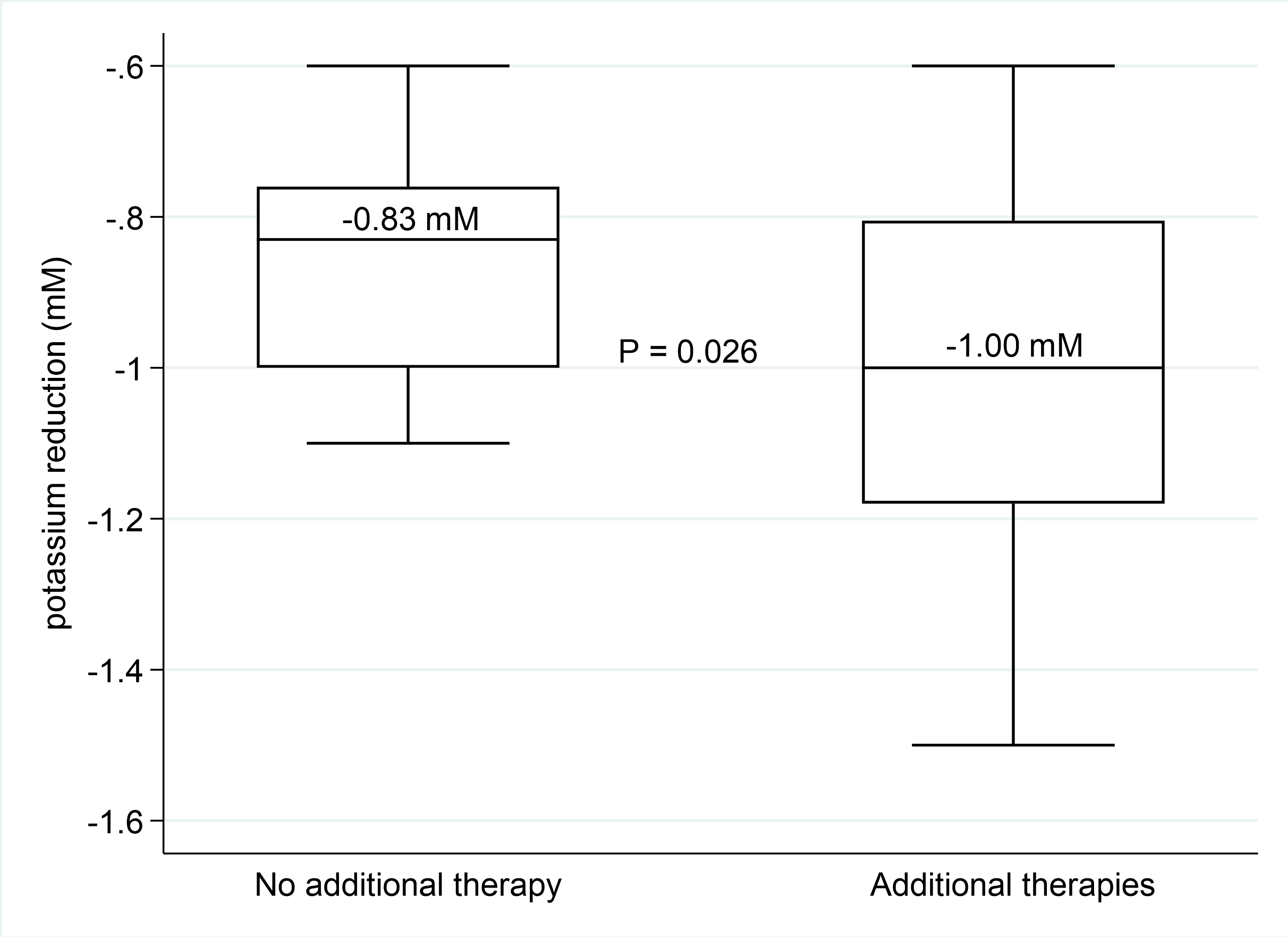

Supplement: S8 Fig — (TIF) [file pone.0268395.s016.tif]
